# Additive Manufacturing, Thermoplastics, CAD Technology, and Reverse Engineering in Orthopedics and Neurosurgery–Applications to Preventions and Treatment of Infections

**DOI:** 10.3390/antibiotics14060565

**Published:** 2025-05-31

**Authors:** Gabriel Burato Ortis, Franco Camargo Zapparoli, Leticia Ramos Dantas, Paula Hansen Suss, Jamil Faissal Soni, Celso Júnio Aguiar Mendonça, Gustavo Henrique Loesch, Maíra de Mayo Oliveira Nogueira Loesch, Felipe Francisco Tuon

**Affiliations:** 1 Laboratory of Emerging Infectious Diseases, Pontifícia Universidade Católica do Paraná, Curitiba 80215-901, PR, Brazilfranco.zapparoli@pucpr.edu.br (F.C.Z.); jamil.soni@pucpr.br (J.F.S.); 2 Unidade do Sistema Neuro-Músculo-Esquelético-UNME, Complexo Hospital de Clínicas da Universidade Federal do Paraná—CHC UFPR, Curitiba 80060-900, PR, Brazil; cjamendonca@yahoo.com.br; 3 Department of Health Education, Florida Christian University, Orlando, FL 32819, USAmaira.loesch@gmail.com (M.d.M.O.N.L.)

**Keywords:** polyetheretherketone, polylactic acid, implants, infection, infection prevention, reverse engineering

## Abstract

The increasing demand for orthopedic and neurosurgical implants has driven advancements in biomaterials, additive manufacturing, and antimicrobial strategies. With an increasingly aging population, and a high incidence of orthopedic trauma in developing countries, the need for effective, biocompatible, and infection-resistant implants is more critical than ever. This review explores the role of polymers in 3D printing for medical applications, focusing on their use in orthopedic and neurosurgical implants. Polylactic acid (PLA), polycaprolactone (PCL), and polyetheretherketone (PEEK) have gained attention due to their biocompatibility, mechanical properties, and potential for antimicrobial modifications. A major challenge in implantology is the risk of periprosthetic joint infections (PJI) and surgical site infections (SSI). Current strategies, such as antibiotic-loaded polymethylmethacrylate (PMMA) spacers and bioactive coatings, aim to reduce infection rates, but limitations remain. Additive manufacturing enables the creation of customized implants with tailored porosity for enhanced osseointegration while allowing for the incorporation of antimicrobial agents. Future perspectives include the integration of artificial intelligence for implant design, nanotechnology for smart coatings, and bioresorbable scaffolds for improved bone regeneration. Advancing these technologies will lead to more efficient, cost-effective, and patient-specific solutions, ultimately reducing infection rates and improving long-term clinical outcomes.

## 1. Introduction

The global population is following a trend of demographic aging, aligning with worldwide statistics. In other words, life expectancy is increasing and, consequently, the prevalence of degenerative diseases is rising at the same rate. In an effort to restore motor functions and relieve pain, arthroplasties have become increasingly frequent treatment options [1]. At the other extreme, many underdeveloped countries have a large population of young adults, a deficient public transportation system, vast metropolitan areas, and a low level of education [2]. These factors culminate in a rising number of orthopedic traumas and severe polytraumas, such as traumatic brain injuries (TBI) [3]. Arthroplasty is a procedure that aids in mobility, joint discomfort, and pain relief for patients with various musculoskeletal disorders, with advanced age being one of the most common reasons for undergoing the procedure [4].

Among arthroplasties—whether of the hip, knee, or shoulder—it is estimated that between 1% and 3% of the procedures will develop acute or late infections, leading to the removal of the prosthesis and its replacement with spacers [5]. In cases of periprosthetic joint infection (PJI), an additional surgical intervention is required, which may involve removing the implant and inserting a temporary antibiotic-loaded spacer. This spacer occupies the space left by the removed prosthesis, maintaining limb stability and alignment while releasing antibiotics to treat the infection. Typically made of polymethylmethacrylate (PMMA), spacers can be commercially available or custom-made by the surgeon. Due to the high cost of commercial spacers, many are handcrafted, which increases surgical time, the risk of bleeding, reoperations, spacer breakage, and reduces functionality [6]. Currently, different metals and polymers have been tested as new materials for implants. Not only have the biomechanical differences been evaluated, but the surface characteristics and the biofilm risk formation are features we need to understand.

In this review, we will discuss the various aspects of polymers for 3D printing in the context of orthopedic and neurosurgical implants, expanding the focus of these materials to additive manufacturing with antimicrobial agents.

## 2. Epidemiology of Orthopedical Implant Infections and Cranioplasty

Globally, it is estimated that between 35 and 40 million people require prosthetic and orthotic services—a number expected to rise significantly in the coming decades due to population growth, increased life expectancy, and the growing prevalence of non-communicable diseases, such as diabetes and stroke. The World Health Organization warns that only 5% to 15% of individuals who could benefit from these assistive devices actually have access to them, even in high-income countries. Furthermore, the lack of public policies, adequate funding, and trained professionals severely limits the availability of these services in many regions. The WHO emphasizes that investment in prosthetic and orthotic services should not be regarded merely as a cost, but as a strategy to promote social inclusion and economic return, which allow users to regain autonomy, re-enter the workforce, and reduce their dependence on health and social care systems [7].

Surgical site infection (SSI) remains one of the most common complications following surgical procedures worldwide, affecting up to one-third of the patients in low- and middle-income countries (LMICs). According to the World Health Organization, the average incidence in these settings is 11.8 per 100 surgical procedures, with reported rates ranging from 1.2% to 23.6%. In high-income countries, although the prevalence is lower, SSIs remain the second most frequent type of health care-associated infection. In Europe, data from the European Centre for Disease Prevention and Control indicate infection rates of 9.5% for colon surgery, 3.5% for coronary artery bypass grafting, 2.9% following cesarean section, and 1.0% after total hip arthroplasty. These figures highlight a persistent global challenge, despite advances in aseptic techniques and prophylactic antibiotic use [1]. In the United States, approximately 1 million hip and knee arthroplasties are performed annually, with a significant increase in revision surgeries. Between 2005 and 2030, the total number of procedures is expected to surpass 4 million. Despite improvements in aseptic conditions and antibiotic prophylaxis, infection rates following arthroplasties are rising globally. A study conducted in California reported infection rates of 2.3% after total hip arthroplasties and 2% for knee arthroplasties. In Portugal, data from a study at Hospital de Santo António indicated that the average cost of an aseptic revision is more than three times that of a primary arthroplasty without complications, and 1.5 times higher than the cost of revisions due to non-infectious causes [8].

Surgical site infections (SSIs) are among the most frequent complications following medical procedures in healthcare facilities. They contribute to prolonged hospital stays, increased hospitalization costs, morbidity, and mortality [9]. The infection rate following knee prosthesis implantation ranges from 0.8% to 1.9%, while, for total hip prostheses, it varies between 0.3% and 1.7% [9]. The mortality associated with prosthetic infections in elderly patients is estimated to be between 5% and 10% of cases [10].

Infections are directly linked to prosthetic contamination, which may occur during prosthesis handling in the operating room due to the presence of pathogens resulting from inadequate hygiene, poor asepsis at the surgical site, improperly sterilized equipment, inadequate personal hygiene of the medical team, or incorrect pre-surgical prosthesis cleaning [11]. Contamination results from multiple factors, including bacterial elements, prosthetic material, and host-related factors. Bacterial adhesion to prosthetic surfaces depends on the material used, influenced by smoothness and porosity [11].

Several factors increase the risk of infection in surgical patients, including comorbidities, such as obesity, poor nutritional status, rheumatoid arthritis, and diabetes mellitus, in addition to superficial wound complications. Obesity is considered a significant risk factor as it prolongs surgical duration and affects prosthesis longevity. Rheumatoid arthritis patients have a higher infection risk due to immunosuppressive medication use, while diabetes increases susceptibility by promoting biofilm formation due to high glucose levels [8]. In England, data from the National Surveillance Program for Surgical Site Infections (SSISS) show that, among 72,000 patients undergoing hip and knee replacement surgeries in 2010/11, 1221 infections were recorded, with 85% occurring within the first 30 days, and an average infection onset time of 14 days [2]. Of these infections, 42% were superficial, 43% were deep incisional, and 15% involved organs or spacers, with superficial infections limited to the initial postoperative period. By contrast, long-term follow-up data from the U.S. indicate that nearly one-fourth of infections occur between two- and ten-years post-surgery, highlighting an ongoing risk beyond the first year [8].

Between 2010 and 2011, 1221 infections were recorded among 72,000 patients undergoing hip and knee replacement procedures. According to hospital monitoring, readmissions, and post-discharge follow-ups, most infections (85%) occurred within the first 30 days, with an average infection onset of 14 days [4]. Only a portion of post-surgical infections are identified through microbiological sampling, leading to the identification of a specific pathogen. Including non-laboratory-confirmed infections is crucial for providing a comprehensive and comparable measure of incidence, especially given potential variations in sampling practices across hospitals. Understanding pathogen distribution is fundamental for guiding local and national prevention strategies, including selecting appropriate prophylactic antibiotics [4].

Studies analyzing causative organisms indicate that *Staphylococcus aureus* is the most common pathogen in SSIs among patients undergoing hip and knee arthroplasties. National surveillance data from England in 2010/11 revealed that 44% of microbiologically confirmed SSIs within a year after surgery were attributed to *S. aureus*, with 20% of cases being methicillin resistant. Coagulase-negative staphylococci were also significant, accounting for 31% of infections, while *Enterococcus* species caused 12%. The remaining infections were caused by *Escherichia coli*, *Enterobacter* spp., *Pseudomonas* spp., and streptococci (7% each), with 28% of cases being polymicrobial [4].

Monitoring changes in SSI etiology has demonstrated the impact of methicillin-resistant *S. aureus* (MRSA) screening and decolonization policies introduced in England, initially targeting high-risk groups in 2006, and later expanding to all patients in 2009. Before 2006, between 25% and 30% of hip/knee SSI cases were caused by MRSA; by 2010/11, this proportion had decreased to 8%. These pre-admission screening policies, alongside other interventions, likely contributed to a significant reduction in invasive MRSA infection rates in English hospitals [4]. The primary causes of post-arthroplasty SSIs (periprosthetic joint infections, PJIs) are microbial adhesion and biofilm formation, responsible for 60% of hospital-acquired infections. Biofilm-related infections can lead to tissue destruction, systemic pathogen transmission, severe disease, and even death. Bacterial adhesion, a critical factor in implant-associated infections, is influenced by bacterial hydrophobicity and charge, implant surface characteristics, environmental conditions, blood tissue responses, and specific adhesion molecules. A biofilm is an organized microbial colony adhering to surfaces through a polymeric matrix composed of polysaccharides, proteins, and DNA. The biofilm formation process involves several stages: initial cell attachment, adhesion, matrix development, biofilm maturation, and dispersion, which allows new biofilms to form in other areas [4].

A study published in the Brazilian Journal of Nephrology evaluated the use of sonication as a method for recovering microorganisms from biofilms on urinary catheters in critically ill patients admitted to intensive care units (ICUs). When compared to conventional urine culture, sonication demonstrated higher effectiveness, yielding a positivity rate of 13.8%, in contrast to only 3.4% observed with standard cultures. Microbiological analysis of the sonicated catheters revealed the enhanced detection of microorganisms, including anaerobes, suggesting that sonication is a valuable tool for identifying infections associated with urinary devices, particularly those involving microbial biofilms [12].

Sonication for the detection of microorganisms on orthopedic devices with in vitro biofilms was evaluated using several processing methods, including matrix-assisted laser desorption/ionization time-of-flight mass spectrometry (MALDI-TOF MS) and real-time quantitative polymerase chain reaction (qPCR). The results indicated that sonication is highly effective in the recovery of microorganisms from biofilms. Furthermore, qPCR demonstrated greater sensitivity in the detection of bacterial DNA, including in samples with negative culture results [13].

A qPCR was able to detect bacterial DNA in all analyzed samples, including those with negative culture results, demonstrating superior sensitivity, particularly in patients who had received prior antibiotic treatment. However, the authors caution that a qPCR may overestimate the presence of infection, as it can detect DNA from non-viable bacteria. Therefore, its use is recommended as a complementary method to conventional microbiological culture in the diagnosis of infections associated with orthopedic implants [14].

## 3. Current Landscape of Orthopedic Implants

Over the past decades, orthopedic implants have undergone significant evolution, driven by remarkable advances in software, biomaterials, innovative surface treatment technologies, and increasingly sophisticated manufacturing techniques. The selection of materials for these implants is crucial, as it requires careful consideration of various mechanical and biological properties, including friction and wear. These factors are essential not only for ensuring biocompatibility but for the long-term durability of the implants, guaranteeing that they can perform effectively and safely throughout their expected service life [15].

Materials commonly used in the fabrication of orthopedic implants encompass a wide range of options (Table 1). Notably, stainless steel alloys (ASI316L) are recognized for their durability and corrosion resistance; chromium–cobalt–molybdenum (CCM) alloys provide excellent wear resistance; and titanium alloys (Ti_6_Al_4_V) are prized for their lightweight nature and biocompatibility. In addition to these metallic alloys, other materials such as ceramics—valued for their high compressive strength and chemical inertness—polyethylene, known for its low-friction properties in joint applications, and polymethylmethacrylate (PMMA) bone cement, widely used for prosthetic fixation due to its gap-filling capability and rapid polymerization, are frequently employed. Each material presents its own set of advantages and disadvantages that must be carefully considered, including factors such as mechanical strength (critical for withstanding bodily loads), chemical stability (which determines durability in the biological environment), and potential toxicity, a key concern for avoiding adverse reactions in surrounding tissues [16].

The surfaces of medical devices play a decisive role in the host’s biological response and in implant performance. These surfaces are reactive and continuously seek to minimize their reactivity through ongoing processes of adsorption, restructuring, and chemical reactions that constantly modify their properties. Such dynamic changes directly influence the interaction between the implant and the surrounding tissues, potentially affecting both bone integration and the risk of infection [16].

Currently, various modifications are being applied to the surfaces of orthopedic devices with the aim of promoting favorable responses to both acute and chronic infections in host tissue, reducing local inflammation at the implant site, enhancing implant integration with bone tissue, and lowering the risk of infection [17]. Emerging technologies, such as 3D printing and nanotechnology, are being explored for the development of personalized implants and for improving osseointegration [15]. Alongside these innovations, the use of synthetic bone substitutes has gained increasing popularity. These substitutes offer several advantages: they can be produced in unlimited quantities, molded to fit bone defects precisely and, thanks to advances in materials engineering, are now available in forms that mimic the mechanical and biological properties of natural bone. These substitutes feature porous structures, biocompatibility, bioabsorbability, and promote both osteoconduction and osseointegration [18,19].

Biofilm formation is a significant concern in orthopedic implants, as it contributes to chronic infections that are often resistant to antibiotics and host immune responses [20]. The propensity for bacterial adhesion and biofilm development varies substantially across different implant materials. Stainless steel and cobalt–chromium alloys exhibit relatively high levels of biofilm formation due to their surface roughness and lower biocompatibility [21]. By contrast, titanium and its alloys, particularly Ti6Al4V, tend to exhibit reduced bacterial adhesion and biofilm development, largely attributed to their favorable surface energy and oxide layer, which promotes biocompatibility and reduces microbial colonization [22].

Ceramics, due to their chemical inertness and smooth surfaces, generally show lower levels of biofilm formation compared to metals; however, their brittleness limits their broader use. Polyethylene, commonly used in the articulating surfaces of joint prostheses, can support biofilm formation, especially when its surface is degraded or damaged. Polymethylmethacrylate (PMMA), while widely used as bone cement, is particularly susceptible to bacterial colonization due to its porosity and surface irregularities post-polymerization [23,24].

## 4. CAD Technology

**Figure 1:**
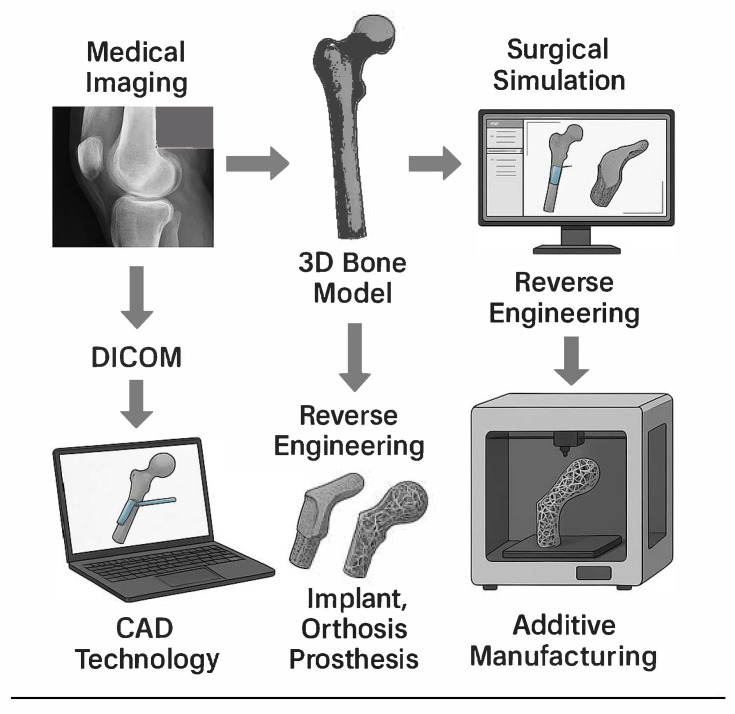
Concept of reverse engineering in prosthetic joint infections with potential use of additive manufacturing with antibiotics.

The use of medical imaging in the additive manufacturing (AM) process applied to medicine is directed toward teaching, planning, training, surgical simulation, and the development and manufacturing of instruments and implants. By employing DICOM (Digital Imaging and Communications in Medicine) image exams—such as computed tomography (CT)—it is possible to reproduce three-dimensional bone structure. Once the 3D bone model is created, surgical simulations can be carried out in a CAD (computer-aided design) environment, accurately modeling instruments and bone implants to adapt to both the surface and interior of bones. This technology enables the development of implant, orthosis, and prosthesis designs with the mechanical and topological optimization of prototypes, while reducing the overall time and resources expended in the process (Figure 1).

In the development of orthopedic implants, a particularly useful tool is reverse engineering. This industrial process is employed to improve an object by studying its geometry inside and out, as well as its structure, operation, and characteristics. From this initial examination, a “clone” of the original object is produced to study and enhance it. In medicine, this approach is used to reproduce implants, prostheses, and orthoses with improved adaptation to anatomical details. By integrating reverse engineering with novel medical imaging acquisition technologies, new 3D printing methods, and advanced CAD software, it becomes possible to plan treatments for bone defects that will be reconstructed in the geometric modeling of prostheses and implants [25,26].

## 5. The Use of Additive Manufacturing for 3D Printing in Medicine

Additive manufacturing (AM) for 3D printing is a technology that enables the creation of three-dimensional objects from a digital model. This manufacturing process does not require molds for production; instead, objects are fabricated layer-by-layer, reducing material waste and minimizing environmental impact. According to the literature, this process can save up to 70% of raw materials, and reduce production time by up to 30%. Compared to traditional subtractive manufacturing, AM offers a faster and more cost-effective method for producing complex shapes. Additionally, it allows for high levels of customization and facilitates on-demand production of replacement parts and custom components, reducing the need for large inventory stocks [27]. This technology has found applications in a wide range of industries, including medicine, architecture, automotive, and aerospace, among others. In medicine, for example, 3D printing is used to create personalized prostheses, medical implants, and anatomical models for surgical planning [28].

According to Mendonça et al. (2024), AM technology has been employed to enhance and develop new materials and designs for orthopedic implants, with a growing role in the manufacturing process [29]. Tilton et al. (2021) suggested that AM is a prime candidate to become the next generation of orthopedic implant design and manufacturing [30]. These authors have highlighted that, compared to traditional manufacturing methods, AM provides unique advantages in economically producing small-volume batches of highly complex products. Since AM does not require tool-dependent designs and materials (e.g., jigs and instruments), it enables the production of customized implants with various specifications, making non-conventional implants more economically viable as production scales up. Furthermore, the design freedom offered by AM allows for the easy integration of porous structures, which promote bone growth and the biological fixation of implants [31].

The use of customized prostheses through 3D printing has positively transformed surgical practice, particularly in fields such as reconstructive plastic surgery. By enabling the fabrication of patient-specific implants based on medical imaging, this technology ensures precise anatomical fit, enhances both aesthetic and functional outcomes, and reduces operative time. Furthermore, customization helps to minimize intraoperative complications and infection risks by reducing the need for adjustments during the procedure. The capability to produce these prostheses directly within the hospital setting represents a significant advancement in personalized medicine, making treatments more efficient, safer, and better tailored to the specific needs of each patient [32].

The integration of 3D printing technologies within hospital environments, focusing on their potential to mitigate bacterial contamination and to enhance additive manufacturing processes for medical applications. Materials commonly used in medical 3D printing were assessed for their susceptibility to microbial colonization. The results have demonstrated that antimicrobial-enhanced filaments containing copper nanoparticles and silver ions significantly reduced bacterial growth compared to conventional PLA. These findings underscore the critical importance of selecting appropriate materials and implementing rigorous hygiene procedures in the clinical deployment of 3D-printed devices. Properly improved and managed, in-hospital 3D printing offers a viable, customizable, and timely approach to producing medical devices, contributing to patient safety and operational efficiency in healthcare settings [33].

## 6. Thermoplastics Used in the Healthcare Industry

Polylactic acid (PLA) is widely used due to its biocompatibility, allowing for safe contact with living tissues without causing adverse reactions. Its biodegradability is environmentally beneficial, as it decomposes naturally, reducing environmental impact. The mechanical properties of PLA provide strength and durability, making it suitable for various applications, from temporary sutures to permanent orthopedic implants. Additionally, PLA is non-toxic, even in its degraded form, ensuring safety in long-term biomedical applications [34]. PLA is a biodegradable thermoplastic polymer widely used in 3D-printed medical devices. However, PLA surfaces are susceptible to biofilm formation, which can compromise device functionality and patient health [35]. Studies have shown that the surface roughness and hydrophobicity of PLA significantly influence bacterial adhesion and subsequent biofilm development [36].

Among biodegradable polymers, poly(L-lactic acid) (PLLA) is highly valued for fracture fixation, tissue repair, and tissue engineering due to its high mechanical strength. However, its degradation byproducts create an acidic environment that may lead to inflammatory responses. Moreover, its limited bioactivity often requires the addition of bioactive ceramics, which restricts its clinical applications [37].

Poly(glycolide-co-lactide) (PLGA) is widely employed in orthopedic implants due to its biodegradable and biocompatible properties. This copolymer consists of polylactic acid (PLA) and polyglycolic acid (PGA), with adjustable ratios that allow for control over degradation rates. This characteristic makes PLGA highly versatile for various medical applications. However, despite its biocompatibility, the clinical use of pure PLGA in bone regeneration is limited due to its low osteoconductivity and insufficient mechanical properties for load-bearing applications [38].

PETG is obtained through glycol modification, which reduces the crystallinity of polyethylene terephthalate (PET) while preserving its biological properties. Although PET exhibits good mechanical properties and biocompatibility, its high crystallinity limits its printability. The modification to PETG results in a material with great potential for human tissue engineering applications [39].

Polyethylene terephthalate glycol is a thermoplastic polymer widely utilized in medical and dental applications; however, its susceptibility to biofilm formation presents clinical challenges. In vitro studies have demonstrated that PETG surfaces can support the adhesion and growth of various microorganisms, including *Streptococcus mutans*, *Streptococcus sanguinis*, *Staphylococcus aureus*, *Staphylococcus epidermidis*, *Lactobacillus casei*, and *Candida albicans* [40]. The surface roughness of PETG materials has been identified as a contributing factor to increased bacterial adhesion and biofilm accumulation. Therefore, strategies aimed at modifying the surface properties of PETG, such as polishing or applying antimicrobial coatings, are being explored to mitigate biofilm-related risks and enhance the material’s suitability for long-term clinical use [41].

Developed in the 1980s, polyetheretherketone (PEEK) gained prominence as a thermoplastic polymer, particularly in orthopedic and trauma applications since the 1990s. PEEK is highly biocompatible and exhibits favorable mechanical properties. However, its inert surface may facilitate bacterial colonization and biofilm formation. It is widely used in bone implants due to its mechanical properties, including an elastic modulus like human bone, biocompatibility, and chemical stability. However, its bioinertness limits its clinical applications, as it does not effectively promote osseointegration [42,43]. Polyetheretherketone is a high-performance thermoplastic increasingly utilized in medical devices due to its favorable mechanical properties and biocompatibility. However, its susceptibility to biofilm formation poses significant clinical challenges. In vivo studies have demonstrated that PEEK surfaces can support biofilm development by oral bacteria, with levels comparable to or exceeding those on titanium and zirconia surfaces [44]. The accumulation of biofilms on PEEK implants can lead to complications, such as peri-implantitis and implant failure. To mitigate these risks, various surface modification strategies have been explored, including sulfonation, incorporation of bioactive agents, and application of antimicrobial coatings [45]. These approaches aim to reduce bacterial adhesion and biofilm formation on PEEK surfaces, enhancing the material’s suitability for long-term clinical applications.

Thermoplastic polyurethane (TPU) has multiple applications in orthopedic implants due to its mechanical properties and biocompatibility. TPU is used in biomedical materials designed for low-temperature applications due to its shape-memory properties. The research has indicated that TPU with appropriately rigid segments can be molded at low temperatures (36–46 °C), enabling the production of customized orthotic devices, such as wrist braces, without causing cytotoxicity. TPU also plays a crucial role in 3D printing for biomedical applications, allowing for the creation of implants with complex geometries and internal architectures that meet precise clinical requirements. TPU produced through 3D printing has demonstrated good cell viability and tissue integration, showing promise for the development of personalized medical implants [46,47].

Biofilm formation on TPU poses a significant challenge in clinical settings. Its surface characteristics can facilitate microbial adhesion and subsequent biofilm development [48]. Studies have demonstrated that pathogens, like *S. aureus* and *S. epidermidis*, readily form biofilms on TPU surfaces, leading to persistent infections that are difficult to treat due to the protective nature of the biofilm matrix [49]. The presence of biofilms not only complicates treatment but increases the risk of device failure and patient morbidity. To mitigate these risks, the research has focused on modifying polyurethane surfaces to resist biofilm formation. For instance, coating polyurethane with diamond-like carbon has been shown to reduce bacterial adhesion and biofilm development [50].

Polycaprolactone (PCL) is an aliphatic polyester widely used in bone tissue engineering. In vivo studies with PCL scaffolds applied to a critical calvarial defect treatment in rats have demonstrated that these scaffolds support new tissue formation. However, PCL has limited bioactivity, a slow degradation rate, and mechanical properties that are insufficient for load-bearing applications [39]. In the Table 2 we compare different the filaments used in fused filament fabrication for 3D printing in medical devices.

## 7. Polymers and Antimicrobial Action in Medicine

The treatment of periprosthetic joint infections (PJI) depends on the onset time and severity of the infection. Early infections can be treated with debridement, antibiotics, and implant retention, while late-stage infections often require implant removal and a two-stage revision arthroplasty, involving complete prosthesis removal, debridement, and placement of a temporary antibiotic-impregnated spacer. After an appropriate course of intravenous and/or oral antibiotic therapy, the implantation of a new prosthesis is planned [51].

In PJI treatment, both mobile and static spacers can be used. The primary material currently utilized for spacers is polymethylmethacrylate (PMMA). Following the removal of infected components, PMMA is prepared intraoperatively with varying types and amounts of antibiotics [52].

Strategies for preventing and controlling implant-related infections include prophylactic antibiotic administration before surgery and the use of antibiotic-loaded PMMA bone cement. The most commonly used antibiotics in this context are gentamicin, rifampicin, vancomycin, and tobramycin. Additionally, biomaterial surface modifications using coatings, such as photoactive materials, antibiotic-hydroxyapatite, nanostructured coatings, nanosilver, antiseptics, and antimicrobial-impregnated agents, are also crucial approaches. Ensuring that these materials exhibit biocompatibility with the patient is essential [8].

Biocompatibility refers to the ability of biomaterials, whether synthetic or naturally derived, to interact with the human body without causing adverse reactions or harm. It is a crucial prerequisite for the successful use of biomaterials in medical applications. Since these materials are often intended for long-term implants or direct tissue contact, biocompatibility ensures immune system compatibility and promotes healing [53].

A promising alternative for use as an antibiotic carrier and as a spacer material in infected arthroplasties is polylactic acid (PLA). This biomaterial is gaining increasing attention in the clinical field due to its remarkable mechanobiological properties. PLA is widely employed in additive manufacturing (AM) due to its biodegradable and biocompatible nature. Derived from renewable resources, such as corn starch and sugarcane, PLA represents a sustainable alternative for biomedical applications. Extensive studies have demonstrated its biocompatibility, highlighting the absence of cytotoxic effects, further reinforcing its potential for safe use in medical devices, such as prostheses and implants [54].

Synthetic biodegradable polymers used in orthopedic devices have durability carefully tailored to withstand mechanical loads during the bone healing process, ensuring temporary structural integrity. Mechanical degradation occurs in a controlled manner, with a gradual loss of strength as the polymers are metabolized by the body, preventing premature failure. The biocompatibility of these polymers is emphasized, as their degradation produces non-toxic byproducts that minimize inflammatory responses, making them suitable for clinical use without the need for surgical removal [55].

The administration of antibiotics through an antibiotic-impregnated medical device is advantageous as it enables localized and effective drug delivery without causing tissue damage, ensuring controlled antibiotic release. This system is also effective against biofilm formation, as bacteria are unable to adhere to the modified surface [56]. The concept of antibiotic-releasing coatings aims to reduce bacterial adhesion and prevent biofilm formation on implants. In this approach, antimicrobial agents are combined with polymeric materials—either biodegradable or non-biodegradable—and applied to the implant surface using various methods, such as impregnation or adsorption. The main advantage of this technology is the ability to modulate antibiotic release, controlling the rate and duration of drug delivery [56].

The incorporation of antimicrobial compounds into PLA for medical applications primarily aims to confer antibacterial properties to medical devices and biomaterials, thereby reducing the risk of device-associated infections. A common strategy involves modifying the PLA surface with antimicrobial compounds. One study demonstrated that quaternized poly(2-(dimethylamino)ethyl methacrylate) was used to create PLA surfaces with significant antibacterial activity against both Gram-negative and Gram-positive bacterial strains [57].

Polymeric coatings can be produced using various techniques, including dip coating, spin coating, spray coating, and solvent casting. Among these, dip coating is the most widely used method in both laboratories and industry due to its simplicity, low cost, reliability, and reproducibility. This technique allows researchers to efficiently adjust the processing parameters. The thickness of the generated films can be controlled by adjusting the immersion duration, the withdrawal speed, and the rheological properties of the coating solution. One study demonstrated that vancomycin-impregnated PLLA coatings were capable of reducing bacterial populations by approximately 80–90% [56].

Another technique involves extruding thermoplastic filaments pre-mixed with the antibiotic or treatment compound. When the filament is processed in a 3D printer, the printed object is already impregnated with the active agent. PLA has relatively low crystallinity, which may hinder its processing; however, the insertion of branched structures in its chains can enhance crystallization and improve processing efficiency. A study evaluating the inclusion of additives in PLA modified with maleic anhydride (PLA-g-MA) and lysine (PLA-g-Lys) observed that this combination accelerated the crystallization kinetics and improved the 3D printability without compromising the mechanical properties. The presence of these additives influenced crystal formation, altering the melting temperature and degree of crystallinity, with lysine exhibiting a more pronounced effect due to its branched structure. Additionally, the grafting process reduced its molecular weight, increasing chain mobility, and promoting crystallization [58]. A recent study applied an ionic silver coating onto PEEK using a hydroxyapatite film. The modified material exhibited strong bactericidal activity in preventing biofilm formation in both in vitro and in vivo models [43].

The ISO 10993-1:2018 standard [59] outlines the principles for the biological evaluation of medical devices as an integral part of a risk management process, in accordance with ISO 14971 [60]. Aimed at protecting human health, the standard guides the selection of appropriate tests based on the nature and duration of the device contact with the body, promoting the use of existing data and alternative methods to animal testing whenever feasible. It includes a table of recommended biological tests, such as cytotoxicity, sensitization, irritation or intracutaneous reactivity, acute systemic toxicity, subchronic and chronic toxicity, genotoxicity, carcinogenicity, reproductive and developmental toxicity, as well as degradation and implantation studies. The systematic application of these tests is intended to ensure the safety and efficacy of medical devices throughout their life cycle, based on a science-driven and risk-based approach.

The sterilization of biomaterials used in orthopedic implants is a critical step to ensure patient safety and prevent postoperative infections. Different materials require specific sterilization methods depending on their thermal stability, chemical composition, and physical properties. Stainless steel and chromium–cobalt–molybdenum alloys are typically sterilized using steam autoclaving due to their high thermal resistance. Titanium alloys, while also thermally stable, may be sterilized using either autoclaving or low-temperature hydrogen peroxide plasma to preserve surface characteristics important for osseointegration. Ceramics, being chemically inert and heat-resistant, are commonly autoclaved as well. By contrast, polymers such as polyethylene and polymethylmethacrylate (PMMA) are more sensitive to heat; therefore, they are generally sterilized using ethylene oxide gas or gamma irradiation to prevent thermal degradation and preserve their mechanical properties. The selection of an appropriate sterilization technique must balance microbial efficacy with the preservation of material integrity and biocompatibility.

## 8. Application in Cranioplasty and Infection Prevent

In developing countries, factors such as a young population, inadequate public transportation systems, densely populated urban centers, and low educational levels contribute to a high incidence of orthopedic and polytrauma injuries, including traumatic brain injuries (TBI). In Brazil, for example, the incidence rate of TBI is estimated at 65.54 per 100,000 inhabitants [3], likely underestimated, considering that, in the United States, this rate is 300 per 100,000 inhabitants [61].

In severe cases of TBI, often associated with hematomas and cerebral edema, many patients require a surgical procedure known as decompressive craniectomy, which involves the removal of a portion of the skull bone to normalize intracranial pressure. Elevated intracranial pressures, if untreated, can lead to brain death [37]. After the resolution of intracranial hypertension, bone reimplantation is necessary in a procedure called cranioplasty. When a synthetic implant is used, the cost of the material is extremely high, making its use infeasible in public healthcare systems such as the Brazilian Unified Health System (SUS). As a result, autologous bone reuse is often necessary, despite its higher risk of infection compared to synthetic materials [62]. A recent study used PLA for cranioplasty impregnated with vancomycin, which is an important perspective to reduce surgical site infections [29]. Nowadays, PMMA is combined with vancomycin during surgery, but this is not considered an additive at manufacture.

## 9. Conclusions and Future Perspectives

The continuous advancements in biomaterials, additive manufacturing, and antimicrobial surface modifications offer a promising future for orthopedic and neurosurgical implants. The incorporation of 3D printing technology into the development of patient-specific implants allows for enhanced customization, improved biomechanical performance, and reduced surgical risks. Moreover, the ability to integrate antimicrobial agents directly into polymeric and metallic implant surfaces is expected to mitigate the risk of periprosthetic joint infections and surgical site infections, addressing a critical challenge in orthopedic surgery. However, further clinical trials are necessary to validate the long-term effectiveness and safety of these new technologies in real-world applications.

The use of biodegradable polymers, such as polylactic acid and polycaprolactone, represents an innovative approach in regenerative medicine, particularly in the development of bioresorbable implants. These materials offer an alternative to traditional metal implants by reducing long-term complications, such as stress shielding and implant-related infections. The combination of biodegradable scaffolds with bioactive agents, including antibiotics and growth factors, is a promising strategy for improving osseointegration and bone regeneration. Despite these advantages, challenges such as mechanical stability, controlled degradation rates, and long-term biocompatibility need to be addressed through further research and material optimization.

Looking ahead, the integration of nanotechnology, artificial intelligence, and machine learning into the design and manufacturing of orthopedic implants is expected to revolutionize personalized medicine. AI-driven computational modeling can enhance implant design by optimizing material properties and predicting biomechanical behavior. Additionally, smart implants equipped with biosensors may enable the real-time monitoring of infection markers, mechanical loads, and healing progress, thereby improving postoperative outcomes. Future research should focus on developing hybrid implant materials that combine the mechanical strength of metals with the bioactivity of polymers, ultimately advancing the field toward more efficient, cost-effective, and patient-specific solutions for orthopedic and neurosurgical applications.

## Figures and Tables

**Table 1:** Summary of commonly used biomaterials in orthopedic implants, including their composition, main characteristics, advantages, disadvantages, and typical clinical applications.

Material	Composition/Main Characteristics	Advantages	Disadvantages	Common Applications
Stainless Steels (ASI316L)	Alloy of steel with high corrosion resistance	Durability, corrosion resistance	Lower wear resistance compared to other materials	Long-term implants, such as orthopedic screws and plates
Chromium–Cobalt–Molybdenum Alloys (CCM)	Alloys containing chromium, cobalt, and molybdenum	Excellent wear resistance	May be less biocompatible compared to titanium, higher density (heavier)	Joint implants, such as hip and knee prostheses
Titanium Alloys (Ti6Al4V)	Titanium alloy with aluminum and vanadium, known for its light weight and biocompatibility	Light, biocompatible, corrosion-resistant, less prone to immune reactions	Lower wear resistance, higher cost compared to other metals	Bone and joint implants, such as hip, knee, skull prostheses, and orthopedic screws
Ceramics	Inorganic materials with high compressive strength and chemical stability	High compressive strength, chemical inertness, durability in biological environments	Brittle (prone to fractures under impact or shock loads)	Hip implants, bone substitutes, joint components
Polyethylene	Plastic with low friction coefficient, common in joint prostheses	Low friction, wear resistance, durability	Can degrade over time under prolonged use conditions	Components of joint prostheses (e.g., acetabular components in hip prostheses)
Polymethylmethacrylate (PMMA)	Plastic material used for prosthesis fixation, with rapid polymerization	Good bone adhesion, easy to mold, fast curing	Can generate thermal reactions during curing, lacks elasticity and strength compared to other materials	Bone cement, fixation of orthopedic implants, such as in hip and knee prostheses

**Table 2:** Thermoplastics used for prosthetic joint infections with potential use of additive manufacturing with antibiotics.

Material	Key Properties	Medical Applications	Antimicrobial Activity
PLA (Polylactic Acid)	Biodegradable, biocompatible, mechanically durable	Sutures, orthopedic implants, temporary devices	Surface roughness and hydrophobicity increase bacterial adhesion and biofilm development. Lacks intrinsic antimicrobial properties.
PLLA (Poly(L-lactic acid))	High mechanical strength, biodegradable	Fracture fixation, tissue engineering	Similar to PLA, it exhibits no inherent antimicrobial activity. Limited bioactivity may permit bacterial colonization.
PLGA (Poly(glycolide-co-lactide))	Biocompatible, tunable degradation rate	Orthopedic implants, bone regeneration	Lacks inherent antimicrobial properties; susceptible to biofilm formation unless surface-modified.
PETG (Polyethylene Terephthalate Glycol)	Good mechanical properties, biocompatible, printable	Dental and medical devices	Supports adhesion of various microorganisms (e.g., *S. mutans*, *C. albicans*). Surface modifications, like polishing or antimicrobial coatings, are under investigation.
PEEK (Polyetheretherketone)	High strength, elastic modulus similar to bone, biocompatible	Orthopedic and dental implants	Inert surface facilitates bacterial colonization. Biofilms may lead to peri-implantitis. Antimicrobial surface modifications (e.g., sulfonation, bioactive agents) are being explored.
TPU (Thermoplastic Polyurethane)	Flexible, shape-memory behavior, biocompatible, low-temperature processable	Custom orthotic devices, 3D-printed implants	*S. aureus* and *S. epidermidis* readily form biofilms on TPU. Surface treatments, such as diamond-like carbon coatings, are being investigated to mitigate microbial adhesion.
PCL (Polycaprolactone)	Biodegradable, tissue-compatible, easily processable	Bone tissue engineering, scaffolds	Does not exhibit inherent antimicrobial activity. Prolonged presence may facilitate biofilm formation over time.

## Data Availability

Data are available under request.
